# Chinese Herbal Medicine Banxiaxiexin Decoction Treating Diabetic Gastroparesis: A Systematic Review of Randomized Controlled Trials

**DOI:** 10.1155/2013/749495

**Published:** 2013-07-11

**Authors:** Jiaxing Tian, Min Li, Jiangquan Liao, Junling Li, Xiaolin Tong

**Affiliations:** ^1^Department of Endocrinology, Guang'anmen Hospital, China Academy of Chinese Medical Sciences, Beixiange 5, Xicheng District, Beijing 100053, China; ^2^Graduate School, Beijing University of TCM, Beijing 100029, China; ^3^Department of Molecular Biology Research Room, Guang'anmen Hospital, China Academy of Chinese Medical Sciences, Beijing 100053, China; ^4^College of Acupuncture, Beijing University of TCM, Beijing 100029, China

## Abstract

*Objective*. To assess the current clinical evidence of Banxiaxiexin decoction for diabetic gastroparesis (DGP). *Methods*. Electronic databases were searched until December 2012. No language limitations were applied. We included RCTs using Banxiaxiexin decoction/modified Banxiaxiexin decoction for DGP. No restriction for the control group except acupuncture. Applying clinical effective rate as the main outcome index. Data extraction, analyses and quality assessment were conducted according to the Cochrane review standards. *Results*. 16 RCTs involving 1302 patients were finally identified, and the methodological quality was evaluated as generally low. The data showed that the effect of Banxiaxiexin decoction (BXXD) for DGP was superior to the control group (*n* = 1302, RR 1.23, 95% CI 1.17 to 1.29, *Z* = 8.04, *P* < 0.00001). Only one trial recorded adverse events, no obvious adverse event occurred. *Conclusions*. Banxiaxiexin decoction could regain the gastric emptying rate and improve diabetic gastrointestinal symptoms. However, the methodological quality of included studies is low, and long term efficacy and safety are still uncertain, which indicates that the findings above should be read with caution. Thereby, well-designed, large-scale, and high-quality randomized controlled clinical trials with scientific rigor are warranted for stronger evidence in future research.

## 1. Introduction

Diabetic gastroparesis (DGP) is a syndrome characterized by delayed gastric emptying in the absence of mechanical obstruction of the stomach in patients with diabetes, which is a well-established complication of diabetes, first reported in 1958 [1]. The cardinal symptoms include postprandial fullness, nausea, vomiting, and bloating [2, 3]. Symptoms attributable to gastroparesis are reported by 5% to 12% of patients with diabetes [4, 5]. This is consistent with the well-established observation that DGP which typically develops after diabetes mellitus (DM) has been established for ≥10 years [1, 6]. Once established, DGP tends to persist despite the improvement of glycemic control [7], which reduces quality of life on all the main aspects including physical, emotional, mental, social, and body functions [8]. DGP is also associated with higher mortality and morbidity [9]; DM patients with classic symptoms of gastroparesis (including early satiety, postprandial fullness, bloating, abdominal swelling, nausea, vomiting, and retching) and documented delay in gastric emptying are more likely to have cardiovascular disease, hypertension, and retinopathy [10], suggesting that DGP may be related to the complications which are known as complications of poor diabetic control. 

The pathogenesis of DGP has not been clarified. Current pieces of research have found that smooth muscle degeneration in DGP is caused by multiple factors, including autonomic nervous dysfunction, hyperglycemia, gastrointestinal hormone secretion disorder, abnormalities of interstitial cells of Cajal (ICC), and vascular lesions [11–14]. Based on blood glucose control, the available treatment options of modern medical treatment include nutritional support, improvement of gastric emptying using prokinetics, symptom control, and use of a gastric electric stimulator [15, 16]. The increasing number of drugs under development with different mechanisms of action improves clinical symptoms, whereas they are far from clinical satisfaction [17]. Metoclopramide, being one of the typical DGP treatments, has a well-recognized complication which is tardive dyskinesia [18]. Another commonly used drug is Erythromycin, which is a useful agent for short term treatment in hospital; however, its long term benefit is limited due to the development of tachyphylaxis [18]. Besides, the use of botulinum toxin injection and gastric electric stimulator is still controversial [19–21]. The high recurrence rate leads to the further deterioration of the disease [8]. Nowadays, with the incidence of diabetes increasing, more and more people will be perplexed by DGP [22]. Therefore, to seek effective measures of treatment has become a major health problem, which is beneficial to the people's livelihood.

Clinical practice has shown a bright future of traditional Chinese medicine (TCM) in treating diabetes and its complications [23]. Banxiaxiexin decoction (BXXD), a traditional Chinese herbal medicine containing seven commonly used herbs (*Pinellia ternata*, Radix Scutellariae, Rhizoma Zingiberis, Panax ginseng, Radix Glycyrrhizae, *Coptis chinensis*, and Fructus Jujubae), is widely used to treat gastrointestinal discomfort in clinical practice for a long time in China [24–26]. The mechanism of the prescription may be acrid to diffuse and bitter to descend, reinforcing and reducing according to the theory of TCM. A recent research showed that BXXD could improve gastrointestinal motility [27, 28]. Biochemically, BXXD also adds plasma motilin, gastrin, and nitric oxide; suppresses VIP; adjusts gastric myenteric plexus, c-kit positive, and ICC volume; copes against dysrhythmia; and promotes gastric emptying markedly [29–32]. 

According to TCM, diabetic patients are always having dysfunction in stomach and have disorder in ascending and descending. Asthenia and sthenia, cold and heat are mixed up when the course of DM becomes long. According to the theory of TCM, we could use BXXD as an alternative method for treating DGP [33, 34]. There have been numbers of research works indicating that BXXD is effective to DGP, whereas the data supporting the validity is not enough. This systematic review aims to assess the current clinical evidence of BXXD for DGP by conducting the literature reviews in databases for randomized controlled trials (RCTs).

## 2. Methods

### 2.1. Database and Search Strategy

A computer-based online search was conducted in the Medline, Cochrane Library, Chinese Biomedical Literature Database (CBM), Chinese National Knowledge Infrastructure (CNKI), Chinese Scientific Journal Database (VIP) and Wanfang Databases. Search terms used were (“diabetic gastroparesis” OR “gastrointestinal changes” OR “gastrointestinal disease”) AND (“herb” OR “Banxiaxiexin Decoction” OR “BanXia Xie Xin” OR “Banxiaxiexin Tang”) AND (“randomized controlled trial” OR “controlled clinical trial” OR “random” OR “randomly” OR “randomized” OR “control”). We searched all articles published before December, 2012. 

### 2.2. Inclusion Criteria

All the RCTs that used BXXD in treatment group were included. RCTs used BXXD combined with conventional treatment (Domperidone, Mosapride, etc.) compared with conventional treatment were included as well. The study evaluated DGP patients in spite of gender, age, or nationality, but those who had other gastrointestinal diseases were excluded. The main outcome index was clinical effective rate, which was based on the gastric emptying test and gastrointestinal (GI) symptoms variation. The secondary outcome index was FPG. Adverse events would also be measured. Duplicated publications reporting the same groups of participants were excluded.

### 2.3. Data Extraction and Quality Assessment

Data extraction were independently proceeded by two authors (J. X. Tian and M. Li). The extracted data on study included the title of study, authors, year of publication, sample size, gender and age of the participants, name and component of Chinese herbs, details of the control interventions, treatment process, outcomes, adverse effects, and the details of methodological information. Discrepancies were resolved by consensus through discussion between the two authors and, if needed, by asking for the further evaluation of the third party (X. L. Tong). The methodological quality of trials was assessed independently by two authors (J. Q. Liao and J. L. Li) using criteria from the Cochrane Handbook for Systematic Review of Interventions [35]. The items included random sequence generation (selection bias), allocation concealment (selection bias), blinding of participants and personnels (performance bias), blinding of outcome assessment (detection bias), incomplete outcome data (attrition bias), selective reporting (reporting bias), and other biases. We judged each item from three levels (“Yes” for a low risk of bias, “No” for a high risk of bias, and “Unclear” otherwise). Then we assessed the trials and categorized them into three levels: low risk of bias (all the items were in low risk of bias), and high risk of bias (at least one item was in high risk of bias), unclear risk of bias (at least one item was in unclear).

### 2.4. Statistical Analysis

RevMan 5.1 software was used for data analyses, which was offered by Cochrane collaboration. Dichotomous data were expressed as relative risk (RR) and continuous outcomes as weighted mean difference (WMD), both with 95% confidence intervals (CI). Heterogeneity was assessed using the *I* ^2^ test with the significance level set at *I* ^2^ over 50% or *P* < 0.1. If there was no heterogeneity (*I* ^2^ < 50%), we selected the fixed effect model; otherwise we used random effects model in explaining the possible causes of heterogeneity (*I* ^2^ > 50%). Publication bias would be explored by funnel plot analysis if sufficient studies were found [25]. 

## 3. Results

### 3.1. Description of Included Trials

**Figure 1:**
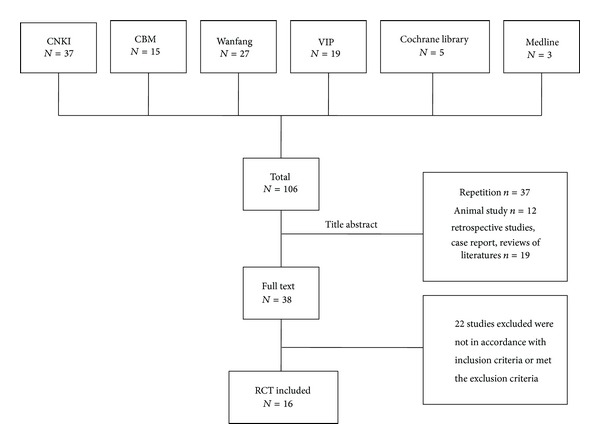
Flow chart of trials selection process.

A total of 106 studies were initially identified, all of them came from electronic database. The search results were summarized in Figure 1. After screening the titles and abstracts, 38 potentially relevant studies were found. Most of them were excluded due to repetitions, retrospective studies, animal study, case report, and reviews of the literature; 37 studies were excluded because of duplicated publication, 12 studies were excluded due to the animal studies, and the rest 19 studies were noncontrolled clinical trials including retrospective studies, case report, reviews of the literature. After a detailed evaluation of full text, 22 studies were excluded, 3 trials claimed that they were RCTs, while they actually were cohort studies using healthy people without intervention as control group, 8 trials were excluded because they only reported the difference after treatment, 6 trials were not evaluated because they included gastric emptying test or gastrointestinal (GI) symptoms, and the intervention of the rest 5 trials was not in accordance with inclusion criteria. Finally, 16 studies, involving 1302 patients, were in accordance with our inclusion criteria and did not met the exclusion criteria. All studies were conducted in China and published in Chinese between 2003 and 2012. The bibliographic details of included studies were given in Table 1.

Among the 16 studies, all participants came from inpatient and/or outpatient department of gastroenterology or endocrinology, and the experimental interventions were oral administration and included 666 males and 636 females. The age of participants ranged from 30 to 80. The diagnosis criteria of research included the following. Eight trails [39, 41, 44, 46–50] mentioned WHO DM diagnosis criteria, acquired certain duration of gastrointestinal discomfort such as postprandial fullness, nausea, vomiting, bloating, and gastrointestinal emptying, and delayed and excluded other gastrointestinal diseases. Four trails [36, 37, 40, 43] mentioned DM diagnosed and certain duration of gastrointestinal discomfort such as postprandial fullness, nausea, vomiting, bloating, and gastrointestinal emptying and delayed and excluded other gastrointestinal diseases. One trail [51] mentioned matching internal diseases diagnose criteria [52], and the rest 3 trials only demonstrated patients with essential DGP.

In the treatment group, there were 13 trails that used herbals alone and 3 trails that used herbals plus conventional western drugs as treatment. Despite the combination of herbals and western medicine in the treatment group, 4 studies used concentrated BXXD and 12 studies used modified BXXD in treatment group. In the control group, all studies used prokinetic medicine alone, 2 of them used Cisapride, and 1 used Mosapride and the others used Domperidone. The period of intervention ranged from 2 weeks to 9 weeks. Three classes were used to evaluate treatment efficacy including significant effective, effective, and ineffective; all trials used clinical effective rate (including significant effective and effective) based on the gastric emptying test and gastrointestinal (GI) symptoms variation to evaluate efficacy, which was considered as the main outcome index. Two studies [49, 51] recorded FPG variation; we regarded it as secondary outcome index in this systematic review. Adverse events would also be measured.

### 3.2. Methodological Quality of Included Trials

The quality assessments were summarized in Table 2. The sample size of included trials varied from 40 to 120 patients; none of the 16 studies reported details for sample size calculations and none was double-blind, placebo controlled study. Three studies described adequate methods of randomization using random number tables [48, 50, 51], the rest 13 studies reporting “randomly allocating” participants as the method of randomization were not described. No trials had clear descriptions of their method of allocation concealment and blinding procedures. All of 16 trials provided patient characteristics and described similarity between comparison groups in baseline, but no trails reported participant losses, which was hard to determine whether these studies had attrition bias. Only 1 trial reported adverse events and 4 trials [40, 48–50] reported followup. The methodological quality of included studies was assessed to be of generally low according to the predefined quality assessment criteria, which indicated that further investigations might influence the confident intervals of this meta-analysis and the result would likely be reversed.

### 3.3. Effect of the Interventions

#### 3.3.1. Clinical Effective Rate

All included studies compared the clinical effective rate between treatment group and control group after intervention, which was based on the variation of gastric emptying test and gastrointestinal (GI) symptoms. Three classes were used to evaluate treatment effects as significant effective, effective, and ineffective. Different studies had similar evaluation standards, and we pooled varies kinds of measurements together to evaluate the general effective rate. Total effective rate was the combination of significant effective and effective rates, which was considered as the main outcome index. Included trials showed homogeneity in the consistency of the trial results (*χ* ^2^ = 9.64, *P* = 0.84, *I* ^2^ = 0%). Thus, fixed effects model should be used for statistical analysis. The treatment group scored significantly higher than the control group (*n* = 1302, RR 1.23, 95% CI 1.17 to 1.29, *Z* = 8.04, *P* < 0.00001).

**Figure 2:**
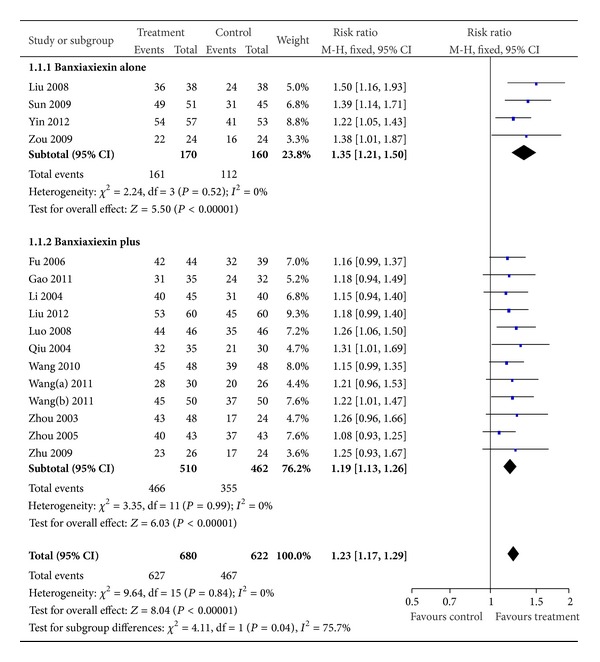
Effective rate comparison between concentrated BXXD and modified BXXD in treatment group.

**Figure 3:**
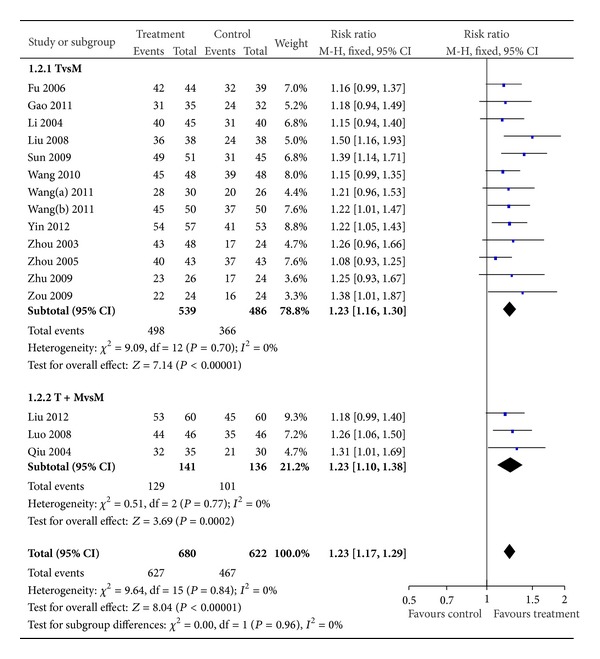
Effective rate comparison between herbals alone and herbals plus conventional western drugs as treatment.

To compare the efficacy of the BXXD with the control group, subgroup analysis had been introduced. Four trails used concentrated BXXD in the treatment group, while 12 trails used modified BXXD. All of the subgroups had shown that treatment group was more effective than control group (*n* = 330, RR 1.35, 95% CI 1.21 to 1.50, *Z* = 5.50, *P* < 0.00001) and (*n* = 972, RR 1.19, 95% CI 1.13 to 1.26, *Z* = 6.03, *P* < 0.00001) (Figure 2). Thirteen studies used herbals alone as treatment group, while 3 studies used herbals plus conventional western drug as treatment. All of the subgroups had shown that treatment group was more effective than control group (*n* = 1025, RR 1.23, 95% CI 1.16 to 1.30, *Z* = 7.14, *P* < 0.00001) and (*n* = 277, RR 1.23, 95% CI 1.10 to 1.38, *Z* = 3.69, *P* < 0.00001) (Figure 3).

#### 3.3.2. Blood Glucose

**Figure 4:**
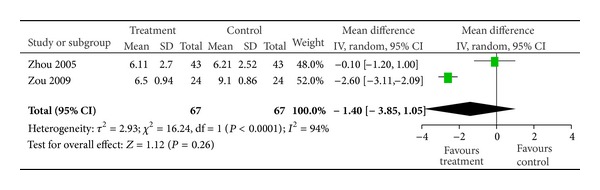
FBG comparison between treatment group and control group.

Two trials provided data for FBG variation [49, 51], and they did not show homogeneity (*χ* ^2^ = 16.24, *P* < 0.0001, *I* ^2^ = 94%). Thus, random effects model should be used for statistical analysis. The meta-analysis of 2 trials showed that there were no significant differences on decreasing FPG between the treatment group and the control group (*n* = 67, MD −1.40, 95% CI from −3.85 to 1.05, *Z* = 1.12, *P* = 0.26) (Figure 4).

### 3.4. Publication Bias

**Figure 5:**
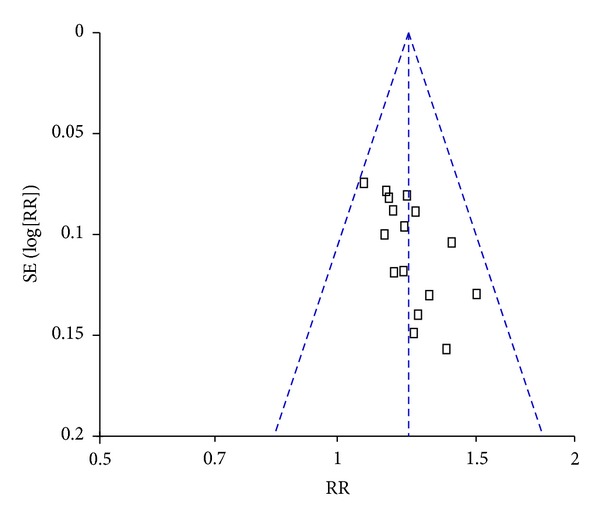
Funnel plot of publication bias.

Funnel plots based on the data of effective rate were elaborated in Figure 5. The figure was asymmetrical, which indicated that potential publication bias might influence the results of this paper. Although we conducted comprehensive searches and tried to avoid bias, since all trials were published in Chinese, we could not exclude potential publication bias.

### 3.5. Adverse Events

Only 1 trail [41] listed safety reports, but no adverse event had been observed in both groups.

### 3.6. Followup

2 trails [48, 50] included followup. Zhou [48] reported 3 recurrences out of 26 patients (11.5%) in treatment group, while 2 out of 6 patients (33.3%) were reported in control group 6 months after intervention stopped. Zhu and Ji [50] reported 3 recurrences out of 14 patients (21.4%) in treatment group, while 2 out of 6 patients (33.3%) were reported in control group 6 months after intervention stopped.

## 4. Discussion

The life quality of those who had diabetic gastroparesis symptoms was severely interfered [9]. Most patients improved glycemic control and symptoms by conventional treatment of western medicine [16, 17]. However, these managements are far from clinical satisfaction [18]. Therefore, it is very important to seek for more safe and effective prevention and treatment. There are researches and clinical trials about TCM treating DGP, including herbs and acupuncture [44, 53, 54]. As BXXD is widely used to treat gastrointestinal discomfort in clinical practice for a long time in China [24–26], we conduct a systematic review to assess the current clinical evidence of BXXD for DGP.

This research is the first systematic review about Chinese herbal medicine treating DGP. In this systematic review, 16 studies involving 1302 participants were included. The review applied clinical effective rate based on the gastric emptying test and gastrointestinal (GI) symptoms variation as the main outcome indexes. The data showed that the effectiveness of BXXD for DGP was superior to the control group. This result is encouraging which indicates new optional treatment for DGP, but the methodological quality of the trials was evaluated generally as low, and the conclusion needs to be confirmed by further study.

The limitations of this review include the following aspects. Though the included researches had detailed including criteria, the participants had years of DM and certain period of gastrointestinal discomfort such as postprandial fullness, nausea, vomiting, bloating, and gastrointestinal emptying delayed; what is worth attention is that there are different appearances in different degrees of DGP. Those who are severely suffering from gastrointestinal symptoms might have serious nausea and vomiting, difficulty swallowing and even could not proceed the gastric emptying test or regular drug taking. Gastric emptying test is a gold standard of DGP diagnosis nowadays, which makes researches of DGP would have limitation in participants. We suggest that it is necessary to accumulate effective treatment for those who are too severe to proceed the gastric emptying test and use vomiting times and duration as auxiliary indicators. It could provide more strong evidence for more widely clinical application.

In this research, the efficacy indicator was effective rate based on the gastric emptying test and syndrome variation. About the gastric emptying test, 5 studies used runtime for determination of efficacy [40, 46, 48–50], while 7 studies used variation level of gastric emptying; the exact runtime was absent [36, 39, 42–44, 47, 51]. One study [45] used gastric emptying residues, and 2 studies [36, 40] used improvement percentage for determination of efficacy. The efficacy determination of DGP is different from blood pressure, lipids, and blood sugar which have explicit numerical index, which makes efficacy determination of gastrointestinal lesions complicated. On the other hand, gastrointestinal discomfort is the most important clinical characteristic. Though the gastric emptying test is very important in efficacy determination as an objective indicator, the variation of gastrointestinal syndromes is also irreplaceable. It corresponds to the fact that judgment of treatment should not be made by only some objective indicators; syndrome improvement is also important to patients. The efficacy determination in the researches included contained evaluation of syndrome variation. But most included trails simply described the syndrome variation. One study [45] used Semiquantitative questionnaire, and the rest 15 studies were lacking unified syndrome questionnaire to evaluate the syndrome variation. Gastroparesis Cardinal Symptom Index (GCSI) is widely used to evaluate the gastrointestinal lesions [55], but none of the included researches used this questionnaire. Thus, it is urgent to standardize the evaluation of gastrointestinal lesions. It could also improve the consistency in future researches.

In those included researches, 2 studies [49, 51] recorded FPG variation, and the meta-analysis showed that there were no significant differences on decreasing FPG between the treatment group and the control group. The rest 14 studies did not mention the variation of blood sugar. We were unable to evaluate potential influences of the blood sugar variation in our analyses. BXXD is used to improve the gastrointestinal dysfunction and restore the normal gastrointestinal peristalsis [28, 29], and research works have proven the mechanisms [30–33]. But few reported the hypoglycemic effect. Though researches have proven the hypoglycemic effect of Coptis Chinensis and Radix Scutellariae, which are contained in BXXD [56–59], whether the whole formula could affect blood sugar and whether the effectiveness in improving gastrointestinal function is related to blood sugar improvement are not clear.

The decocting of BXXD in all included studies was unified twice per day, but the variation among trials was apparent in terms of dosage, treatment course, and sample size. Three studies used combination herbals with prokinetic medicine and, others used herbals alone as treatment. While 4 studies used concentrated Banxiaxiexin decoction the rest used modified. The period of intervention ranged from 2 weeks to 9 weeks. None of them reported sample size calculations, and the efficacy could not be clarified on some outcome measurements due to the small number of studies, thus the reliability of the outcome might be questionable. 

All trials included were lacking description of randomization method; only 3 pieces of research mentioned random form [48, 50, 51], and the other 13 trials just mentioned “randomly allocating” with no detailed information. It is difficult to identify whether those researches proceed randomization adequately. No researches mentioned allocation concealment. Therefore, it may introduce some false “RCTs” in the review and may mislead the results. We have tried to contact the authors for further information about the trials, but regrettably no information provided until now.

No research mentioned blind method which could lead to performance bias and detection bias that patients and researchers were aware of the therapeutic interventions for the subjective outcome measures. The operability of blind method was very low because of using Chinese herbal and western medicine as treatment and control. Only 1 research [41] mentioned safety and described no adverse event after intervention. Even no reports of adverse event and safety should be concerned and recorded in detail. No trails reported participant losses or used intention to treat method, which was hard to determine whether these studies had attrition bias. Only 2 researches [48, 50] mentioned followup. Diabetes gastrointestinal disease is easy to recur, thus it is necessary to proceed a long term followup in research. We tried to avoid language bias and location bias, but all the included researches were published in China; the result was limited in worldwide application. The quality of the methodology was low; future researches should enhance the randomization, safety report, detailed followup, and blind method to improve the quality. In order to explore the efficacy and safety of BXXD treating diabetes gastrointestinal disease, it is urge to proceed more well-designed, complete efficacy indicator, larger scaled, and multiple center randomized clinical trials.

## 5. Conclusion

From this systematic review, we find that BXXD could regain the gastric emptying rate and improve diabetic gastrointestinal symptoms; thus it could be considered as an alternative way to treat DGP. But the efficacy determination system of TCM treating DGP is not established, also the long term efficacy and safety of BXXD treating DGP are still uncertain, the methodological quality is assessed to be of general low and some posible biases exist. The previous results should be read with caution. Thereby, the efficacy determination system of TCM treating DGP should be established soon, and well-designed, large-scale, high-quality randomized controlled clinical trials with scientific rigor are warranted for stronger evidence in the future, while the followup and adverse events should also be clarified in detail. Accumulating clinical evidence of severe gastroparesis is very necessary.

## Figures and Tables

**Table 1:** Characteristics of included RCTs.

Trials	Sample size	Gender	Age (yr)	Intervention			Outcome measure	Balance report of baseline
				Experimental group	Control group	Period		
Fu (2006) [36]	83 (44/39)	(19 M : 25 F)/(17 M : 22 F)	45–65/43–66	Modified Banxiaxiexin decoction (300 mL/d)	Domperidone (10 mg, tid)	9 W	Clinical effective rate Gastric emptying test	Not mentioned

Gao (2011) [37]	67 (35/32)	(21 M : 14 F)/(17 M : 15 F)	34–72 (54)/37–71 (56)	Modified Banxiaxiexin decoction, bid	Domperidone (10 mg, tid)	8 W	Clinical effective rate Gastric emptying test	*P* > 0.05

Li (2004) [38]	85 (45/40)	(28 M : 17 F)/(28 M : 12 F)	50–72	Modified Banxiaxiexin decoction (400 mL/d)	Domperidone (10 mg, tid)	4 W	Clinical effective rate GI symptoms	Not mentioned

Liu et al. (2008) [39]	76 (38/38)	(36 M : 40 F)	58 ± 12	Concentrated Banxiaxiexin decoction, bid	Domperidone (10 mg, tid)	4 W	Clinical effective rate Gastric emptying test	Not mentioned

Liu (2012) [40]	120 (60/60)	(58 M : 62 F)	43.52 ± 25.48	Concentrated Banxiaxiexin decoction, bid plus Domperidone (10 mg, tid)	Domperidone (10 mg, tid)	4 W	Clinical effective rate Gastric emptying test	*P* > 0.05

Luo et al. (2008) [41]	92 (46/46)	(25 M : 21 F)/(24 M : 22 F)	54.3 ± 8.3/54.8 ± 8.6	Modified Banxiaxiexin decoction, bid plus Mosapride (5 mg tid)	Mosapride (5 mg tid)	4 W	Clinical effective rate Gastric emptying test GI symptoms Adverse events	*P* > 0.05

Qiu et al. (2004) [42]	65 (35/30)	(19 M : 16 F)/(17 M : 13 F)	43–75/42–73	Modified Banxiaxiexin decoction, bid plus Cisapride (5 mg, tid)	Cisapride (5 mg, tid)	4 W	Clinical effective rate Gastric emptying test	No significant differences

Sun (2009) [43]	96 (51/45)	(24 M : 27 F)/(18 M : 27 F)	38–69/35–72	Concentrated Banxiaxiexin decoction, bid	Domperidone (20 mg, tid)	2 W	Clinical effective rate Gastric emptying test GI symptoms	*P* > 0.05

Wang (2011) [44]	100 (50/50)	(24 M : 26 F)/(25 M : 25 F)	39–68/38–70	Modified Banxiaxiexin decoction (300 mL/d)	Domperidone (10 mg, tid)	2 W	Clinical effective rate Gastric emptying test GI symptoms	*P* > 0.05

Wang (2011) [45]	56 (30/26)	(18 M : 12 F)/(15 M : 11 F)	54.5 ± 9.6/ 53.8 ± 10.2	Modified Banxiaxiexin decoction (300 mL/d)	Domperidone (10 mg, tid)	4 W	Clinical effective rate Gastric emptying test GI symptoms	*P* > 0.05

Wang (2010) [46]	96 (48/48)	(22 M : 26 F)/(24 M : 24 F)	55 ± 5/ 53 ± 6	Modified Banxiaxiexin decoction (300 mL/d)	Domperidone (10 mg, tid) plus Roxithromycin (150 mg bid)	4 W	Clinical effective rate Gastric emptying test	*P* > 0.05

Yin (2012) [47]	110 (57/53)	(35 M : 22 F)/(30 M : 23 F)	58 ± 10.7/ 59 ± 11.5	Concentrated Banxiaxiexin decoction, bid	Domperidone (10 mg, tid)	4 W	Clinical effective rate Gastric emptying test	*P* > 0.05

Zhou (2003) [48]	72 (48/24)	(20 M : 28 F)/ (11 M : 13 F)	38–64 (56.2)/36–63 (56.1)	Modified Banxiaxiexin decoction, bid	Domperidone (10 mg, tid)	4 W	Clinical effective rate Gastric emptying test	*P* > 0.05

Zhou (2005) [49]	86 (43/43)	(20 M : 23 F)/(19 M : 24 F)	53.01 ± 17.1/ 51.13 ± 18.1	Modified Banxiaxiexin decoction, bid	Cisapride (10 mg, tid)	4 W	Clinical effective rate Gastric emptying test, FBG	*P* > 0.05

Zhu and Ji (2009) [50]	50 (26/24)	(14 M : 12 F)/(11 M : 13 F)	38–64 (56.2)/36–63 (56.1)	Modified Banxiaxiexin decoction, bid	Domperidone (10 mg, tid)	4 W	Clinical effective rate Gastric emptying test	*P* > 0.05

Zou (2009) [51]	48 (24/24)	(14 M : 10 F)/(13 M : 11 F)	54 ± 10.57/ 55.1 ± 10.37	Modified Banxiaxiexin decoction (200 mL/d)	Domperidone (10 mg, tid)	4 W	Clinical effective rate Gastric emptying test, FBG	*P* > 0.05

**Table 2:** Quality assessment of included RCTs.

Trials	Randomization	Allocation concealment	Blinding of participants personnel and outcome assessors	Incomplete outcome data	Selective reporting	Other sources of bias	Risk of bias
Fu (2006) [36]	Unclear	Unclear	Unclear	Yes	No	Unclear	High
Gao (2011) [37]	Unclear	Unclear	Unclear	Yes	No	Unclear	High
Li (2004) [38]	Unclear	Unclear	Unclear	Yes	No	Unclear	High
Liu et al. (2008) [39]	Unclear	Unclear	Unclear	Yes	No	Unclear	High
Liu (2012) [40]	Unclear	Unclear	Unclear	Yes	No	Unclear	High
Luo et al. (2008) [41]	Unclear	Unclear	Unclear	Yes	No	Unclear	High
Qiu et al. (2004) [42]	Unclear	Unclear	Unclear	Yes	No	Unclear	High
Sun (2009) [43]	Unclear	Unclear	Unclear	Yes	No	Unclear	High
Wang (2011) [44]	Unclear	Unclear	Unclear	Yes	No	Unclear	High
Wang (2011) [45]	Unclear	Unclear	Unclear	Yes	No	Unclear	High
Wang (2010) [46]	Unclear	Unclear	Unclear	Yes	No	Unclear	High
Yin (2012) [47]	Unclear	Unclear	Unclear	Yes	No	Unclear	High
Zhou (2003) [48]	Table of random	Unclear	Unclear	Yes	No	Unclear	Unclear
Zhou (2005) [49]	Unclear	Unclear	Unclear	Yes	No	Unclear	High
Zhu and Ji (2009) [50]	Table of random number	Unclear	Unclear	Yes	No	Unclear	Unclear
Zou (2009) [51]	Table of random number	Unclear	Unclear	Yes	No	Unclear	Unclear
